# Porcine Functional Spine Unit in orthopedic research, a systematic scoping review of the methodology

**DOI:** 10.1186/s40634-022-00488-6

**Published:** 2022-06-09

**Authors:** Jacob Hedlund, Lars Ekström, Olof Thoreson

**Affiliations:** 1 Department of Orthopedics, Institute of Clinical Sciences at Sahlgrenska Academy, University of Gothenburg, Gothenburg, Sweden; 2 Orthopaedic Research Unit, Sahlgrenska University Hospital, Mölndal, Sweden; 3 Research and Development Primary Health Care, R&D Centre Gothenburg and Södra Bohuslän, Gothenburg, Sweden

## Abstract

**Purpose:**

The aim of this study was to conduct a systematic scoping review of previous in vitro spine studies that used pig functional spinal units (FSU) as a model to gain an understanding of how different experimental methods are presented in the literature. Research guidelines are often used to achieve high quality in methods, results, and reports, but no research guidelines are available regarding in vitro biomechanical spinal studies.

**Methods:**

A systematic scoping review approach and protocol was used for the study with a systematic search in several data bases combined with an extra author search. The articles were examined in multiple stages by two different authors in a blinded manner. Data was extracted from the included articles and inserted into a previously crafted matrix with multiple variables. The data was analyzed to evaluate study methods and quality and included 70 studies.

**Results:**

The results display that there is a lack of consensus regarding how the material, methods and results are presented. Load type, duration and magnitude were heterogeneous among the studies, but sixty-seven studies (96%) did include compressive load or tension in the testing protocol.

**Conclusions:**

This study concludes that an improvement of reported data in the present field of research is needed. A protocol, modified from the ARRIVE guidelines, regarding enhanced report-structure, that would enable comparison between studies and improve the method quality is presented in the current study. There is also a clear need for a validated quality-assessment template for experimental animal studies.

## Introduction

Many different spinal pathologies can cause back pain but in most cases the cause is still unknown. Further basic research is therefore crucial to gain additional information regarding causal relationship between spinal loads, back pain, and spinal pathologies. Research regarding spinal loading is often done using biomechanical test models [1]. To achieve high research quality, it is vital to validate and in a detailed manner describe the study method. Research guidelines are recommendations on how to ensure high study quality depending on study type. The research guidelines help to minimize unnecessary studies, maximize information published and allow reproducibility and comparability across studies. The ARRIVE (Animal Research: Reporting of In Vivo Experiments) guidelines [2] is a worldwide accepted checklist that support authors of in vivo experimental studies to achieve high quality aspects regarding the study design, method, material, analyzation and report of studies and there are several checklists regarding different in vitro experimental studies, but not any specific for functional spinal units and biomechanical experiments.

Spines from human cadavers and animals are commonly used in varying experimental models for spinal research. Frequently used animals are calves, deer, dogs, goats, pigs, and sheep [3–7]. The porcine lumbar spine resembles the human lumbar spine in both biomechanical properties, load response and tissue structure, and is a well-used experimental model [8–16].

The material and specimen complexes used in biomechanical studies can be of many compositions ranging from a complete spine to small tissue samples from any part of the spine. A Functional Spinal Unit (FSU) consists of an upper and a lower vertebra with an intact intervertebral disc and is an international well-established research model for spine studies. In many biomechanical experimentation settings, the FSU is attached in some way superiorly and inferiorly to a device, which may induce a load on the specimen. The load can be of different vectors/angles, magnitudes/sizes, or a combination of these, and of variable rate and durations depending on study question, method, and protocols [16–18].

There is currently no common consensus regarding the methodology of in vitro spinal experimental biomechanical studies nor an established research presentation guideline, which is why there is a need to conduct a systematic scoping review and present a basic research guideline to achieve comparability, reduce unnecessary experiments and increase study quality.

### Aim

The aim of this study was to conduct a systematic scoping review of previous in vitro biomechanical studies that used porcine functional spinal units (FSU) to gain an understanding of how different experimental methods are presented, summarize the study outcomes, and suggest future reporting guidelines.

## Material and methods

The study methodology was a systematic scoping review [19, 20]. The search inclusion criteria were 1. Pig spine, 2. FSU specimen, 3. Not operated nor instrumented (preparation and testing fixation were accepted), 4. Article published in English language in a peer reviewed journal, 5. No publication date limit.

### Study search protocol and search strategy

A modified version of the Systematic Review Protocol for Animal Intervention Studies (SYRCLE) [21] and the PRISMA-ScR Checklist [22] was used as a general study protocol to ensure systematic approach. The search strategy was a two-phase process: 1. Database search, and 2. Complementary search of first and last author of included studies from phase 1.

Several pilot searches were done according to the inclusion criteria and the final search was done in collaboration with a medical research librarian in the data bases of PubMed, Embase, Cochrane and Web of Science in 2021–04-14. The search protocol: Search ((((((Spine[mh] OR Vertebral Column[tiab] OR Vertebral Columns[tiab] OR Spinal Column[tiab] OR Spinal Columns[tiab] OR Vertebra[tiab] OR Vertebrae[tiab] OR Spine[tiab] OR spinal[tiab])) AND (Mechanical Phenomena[Mesh] OR Biomechanic[tiab] OR Biomechanical[tiab] OR Mechanobiological[tiab] OR Kinematics[tiab])) AND (pig[tiab] OR pigs[tiab] OR piglet[tiab] OR piglets[tiab] OR porcine[tiab] OR porcines[tiab] OR Swine[mh] OR swine[tiab])) AND strength)) NOT (Editorial[ptyp] OR Letter[ptyp] OR Comment[ptyp] OR Case reports[ptyp]) Filters: English, Title, Abstract, Keywords. No letter, comment, editorial.

The complementary author search (phase 2) was done in PubMed and included all primary and last authors from the accepted studies from the database search (phase 1).

**Fig. 1:**
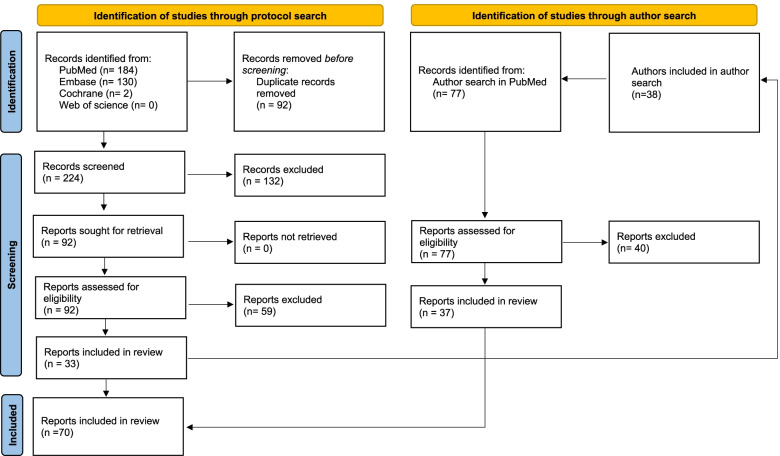
Flowchart of the selection process

The flowchart of the selection method is presented in Fig. 1 [23]. Each abstract was examined by all three authors individually. All abstracts which were considered relevant by two authors were cleared for the next step. The abstracts which were approved by only one author were discussed by all three authors to determine whether they were cleared for inclusion.

The approved articles (*n* = 92) were then read and assessed by the authors. The articles were divided so that each article was read by two of the authors individually. The articles were judged in accordance with the study protocol. Out of the 92 articles that were read, 33 were accepted for data extraction.

All first and last authors of the 33 accepted studies were then included in the complementary author search that involved 38 unique authors. The author search presented an additional 77 new abstracts that were screened according to the previous selection method, and which 37 were accepted for data extraction. In total 70 studies were included in the present study.

### Data extraction

The data relating to the predefined variables were then inserted into previously crafted matrixes (Table 1, 2, 3). Two authors screened the articles individually and compared the data extraction results. If in disagreement or if an uncertainty arose, a second was conducted in collaboration. The variables in the matrices included material type, sample size, mechanical load, test apparatus, study question and outcome of the study.

**Table 1:** List of included studies

Study nr	Year	Journal	Reference
1	2015	Acta of bioengineering and biomechanics	[24]
2	2004	Spine	[25]
3	2005	Clinical biomechanics	[26]
4	2016	Spine Journal	[27]
5	2005	Spine	[17]
6	2005	Clinical biomechanics	[28]
7	2001	Clinical biomechanics	[29]
8	2004	Clinical biomechanics	[30]
9	2008	Spine	[31]
10	2009	Clinical biomechanics	[32]
11	2003	J Orthop Res	[33]
12	2012	Spine	[34]
13	2012	Medical engineering & physics	[35]
14	2001	Clinical biomechanics	[36]
15	2015	The spine journal	[37]
16	2012	Journal of biomechanics	[38]
17	2013	Clinical biomechanics	[39]
18	2013	Medical engineering & physics	[40]
19	2013	Medical engineering & physics	[41]
20	2007	Spine	[42]
21	1998	Spine	[43]
22	2007	Journal of biomechanics	[44]
23	2009	Clinical biomechanics	[45]
24	2005	Spine	[46]
25	2008	Clinical biomechanics	[47]
26	2007	Spine	[48]
27	2010	Knee surgery, sports traumatology, arthroscopy	[18]
28	2006	Spine	[49]
29	2010	European Spine J	[50]
30	2010	Spine	[51]
31	2016	Journal of biomechanics	[52]
32	2020	Journal of biomechanics	[53]
33	2008	Journal of biomechanics	[54]
34	2011	Spine	[55]
35	2002	Journal of biomechanics	[56]
36	2002	Stud Health Technol Inform	[57]
37	2001	Spine	[15]
38	2008	Journal of biomechanics	[58]
39	2005	Spine	[59]
40	2011	BMC Musculoskelet Disord	[60]
41	2001	Journal of biomechanics	[61]
42	1998	Magn Reson Imaging	[62]
43	2019	Journal of biomechanics	[63]
44	2020	Journal of biomechanics	[64]
45	2020	Journal of biomechanics	[65]
46	2019	Ultrasound Med Biol	[66]
47	2020	The Spine Journal	[67]
48	2016	J Biomech Eng	[68]
49	2010	J Biomech Eng	[69]
50	2008	Spine	[70]
51	2015	Journal of biomechanics	[71]
52	2016	Journal of biomechanics	[72]
53	2015	Clinical biomechanics	[73]
54	2005	Clinical biomechanics	[74]
55	2011	Spine	[75]
56	2000	European Spine J	[76]
57	2017	J Experimental Orthop	[77]
58	2016	The Spine Journal	[78]
59	2012	Clinical biomechanics	[27]
60	2013	Journal of biomechanics	[79]
61	2008	Spine	[80]
62	2009	Spine	[81]
63	2010	Clinical biomechanics	[82]
64	1999	J Spinal Disord	[83]
65	1999	Spine	[84]
66	1999	Clinical biomechanics	[85]
67	2017	Med Eng Phys	[86]
68	2014	The Spine Journal	[87]
69	2019	Ergonomics	[88]
70	2018	European Spine J	[89]

**Table 2:** Material information and study apparatus

Study	Level	Breed	Weight	Age	Sample size	Previously frozen	Environmental considerations	Test Equipment
1	Lumbar	na	na	18 months	6	Yes	12 h hydration with phosphate buffer saline solution	Instron 8874
2	Cervical	na	80 kg	6 months	52	Yes	Wrapped in paper wet with saline	Instron 8511
3	Cervical	na	80 kg	6 months	16	Yes	Wrapped in paper wet with saline	Instron 8511
4	Cervical	na	80 kg	6 months	14	na	Heated to body temperature	Instron 8511
5	Lumbar	Domestic	65–73 kg	4 months	16	No, refrigerated	In a plastic bag	MTS Teststar
6	Lumbar	Domestic	~ 80 kg	5 months	24	No, refrigerated	In a plastic bag	MTS Teststar
7	Cervical	na	80 kg	6 months	26	Yes	na	Instron 8511
8	Lumbar	na	na	na	32	na	Contained in plastic sleeve filled with saline	Custom made
9	Lumbar	na	na	na	6	na	na	Instron 8874
10	Cervical	na	na	na	16	Yes	Wrapped in saline-soaked cloth	Instron 8872 custom build
11	Lumbar	na	na	> 16 weeks	6	na	Immersed in an isotonic saline bath cooled to approximately 4 °C	Custom made, 6 DOF
12	Cervical	na	na	na	48	Yes	Moistened with saline every 20 min	Instron 8872
13	Cervical	na	na	na	14	na	Wrapped in saline-soaked gauze	Instron 8872
14	Cervical	na	80 kg	6 months	48	Yes	Wrapped in saline-soaked towel	Instron 8511
15	Lumbar	na	60 kg	na	12	Yes	Sprayed with saline and wrapped in plastic	Custom spine simulator AMTI MC3-A-1000
16	Cervical	na	na	na	96	Yes	na	Instron 8872 custom build
17	Cervical	na	na	na	32	Yes	Wrapped in saline-soaked gauze	Instron 8872 custom build
18	Cervical	na	na	na	30	Yes	na	Instron 8872 Kollmorgen/Danaher Motion AKM23D
19	Cervical	na	na	na	31	Yes	Wrapped in saline-soaked gauze	Instron 8872 Kollmorgen/Danaher Motion AKM23D
20	Thoracic	Domestic	56–61 kg	Immature	14	Yes	Wrapped in moist gauze	Instron 8872
21	Lumbar	Domestic	66 ± 3 kg	Young	12	Yes	na	MTS Teststar
22	Cervical	na	na	na	218	Yes	Wrapped in saline-soaked gauze	Instron 8872
23	Cervical	na	na	na	50	Yes	na	Instron 8872 Kollmorgen/Danaher Motion AKM23D
24	Cervical	na	50–80	na	10	Yes	Wrapped in saline-soaked gauze	Instron 8872
25	Lumbar	Domestic	90–100 kg	8 months	10	Yes	Wrapped in saline-soaked cloth	Instron 8874
26	Cervical	na	Mean 80 kg	Mean 6 months	16	Yes	na	Instron 8511
27	Lumbar	Domestic	65–70 kg	6 months	8	No, refrigerated	Wrapped in saline-soaked gauze	na
28	Lumbar	Domestic	80 kg	Immature	69	Yes	Sprayed with saline	Instron 8872
29	Lumbar	Domestic	mean 78 kg	Mean 7 months	8	Yes	Kept wet by saline-soaked gauze	Instron 8872
30	Cervical	na	~ 80 kg	Mean 6 months	22	Yes	Wrapped in saline-soaked cloth	Instron 8511 custom servo motor
31	Lumbar	na	na	na	9	Yes	Wrapped in saline-soaked gauze	Custom built pendulum design
32	Cervical	na	na	na	22	Yes	na	Pressure transducer (model DPG1000DR)
33	Cervical	na	80 kg	6 months	16	Yes	na	Pressure transducer needle (OrthoAR)
34	Cervical	na	na	6–8 months	20	Yes	Wrapped in saline-soaked gauze	Instron 8872
35	Lumbar	na	na	na	1	na	Circulating isotonic saline at 4C	Custom built load device
36	Lumbar	na	na	> 16 weeks	6	na	Physiological fluid environment	Custom built load device
37	Lumbar	na	na	na	7	na	Room temperature in ambient air	Custom built load device
38	Lumbar	na	na	10 months	8	Yes	Tested in a saline bath at 37 C	Instron 8872
39	Lumbar	na	na	10 months	8	Yes	Tested in a physiologic saline bath (39 °C)	Instron 8872
40	Lumbar	na	na	na	8	na	Wrapped in a saline soaked cloth	Instron 8872
41	Lumbar	na	na	na	6	na	na	na
42	na	na	na	na	1	Yes	na	Custom built load device
43	Cervical	na	na	5–18 months	48	Yes	Encapsulated with plastic-backed saline soaked gauze	Instron 8872 Kollmorgen/Danaher Motion AKM23D
44	Cervical	na	na	na	32	Yes	Temperature-controlled laboratory at 21 °C	Instron 8872 Kollmorgen/Danaher Motion AKM23D
45	Cervical	na	na	na	12	Yes	na	Instron 8872 Kollmorgen/Danaher Motion AKM23D
46	Cervical	na	na	na	24	Yes	Room temperature and surrounded by a water	Instron 8511
47	Cervical	na	na	na	20	Yes	na	Instron 8872
48	Cervical	na	na	na	21	na	Superficial moistening every 20 min	Instron 8872 Kollmorgen/Danaher Motion AKM23D
49	Cervical	na	na	na	30	Yes	Saline soaked cloth wrapped in plastic	Instron 8872 Kollmorgen/Danaher Motion AKM23D
50	Cervical	na	na	na	4	Yes	na	Instron 8511
51	Cervical	na	85 kg	6 months	126	Yes	Hydrated with a saline mist every 15 min	Instron 8872
52	Cervical	na	na	na	14	Yes	Misted with a 0.9% saline solution	Instron 8872
53	Cervical	na	85 kg	6 months	126	Yes	Misted with a saline solution every 15 min	Instron 8872
54	Cervical	na	na	na	18	Yes	Wrapped with saline soaked plastic-backed cloth	Instron 8511 + custom device
55	Cervical	na	na	na	64	Yes	Wrapped in a saline-soaked plastic backed cloth	Instron 8511 + custom device
56	Lumbar	Domestic	55 kg / 195 kg	4 months / 2–3 years	12	Yes	na	MTS Teststar
57	Lumbar	Domestic	75–80 kg	6 months	19	No, refrigerated	Wrapped in saline-soaked gauze	MTS Teststar
58	Cervical	na	80 kg	6 months	30	na	Saline-soaked cloth and plastic wrap	Instron 8511
59	Cervical	na	80 kg	6 months	30	na	Wrapped in cloth soaked in saline along with plastic wrap	Instron 8511
60	Cervical	na	80 kg	6 months	10	na	Saline-soaked cloth and plastic wrap	Instron 8511
61	Lumbar	na	na	6–8 months	5	Yes	Wrapped in a saline soaked towel rehydrated every 20 min	Instron 591 + Instron 8874
62	Cervical	na	80 kg	na	18	Yes	Saline (0.9% NaCl) soaked plastic-backed material and a layer of polythene film	Instron 8511
63	Cervical	na	80 kg	6 months	50	na	Wrapped in a saline soaked cloth and plastic wrap	Instron 8511
64	Cervical	Domestic	80 kg	na	26	Yes	na	Instron 8511
65	Cervical	Domestic	80 kg	6 months	56	Yes	na	Instron 8511
66	Cervical	na	80 kg	na	na	Yes	na	Instron 8511
67	Lumbar	na	na	na	1	Yes	Sprayed and wrapped in paper towel soaked with 0.9% saline solution, triple sealed in plastic bag	Dynamic six-axis spine simulator, dSPACE Ltd
68	Lumbar	Organically farmed pig	60 kg	8–12 months	1	No	wrapped in plastic film at room temperature (20 C)	Zwick 25–200
69	Cervical	na	na	na	48	Yes	3% weight/volume saline soaked tissue	Instron 8872 Kollmorgen/Danaher Motion AKM23D
70	Cervical	na	60 kg	na	28	Yes	na	pressure transducer, model DPG1000DR; 2000 PSIG transducer

**Table 3:** Load protocols

Study	Pre-load	Compression	Flexion	Extension	Lateral bending	Rotation	Shear	Combined	Angle	Rate	Duration	Load Magnitude	Mechanical properties reported
1	1 mm	Yes	No	No	No	No	No	No	Na	na	10 s	na	Disc pressure 0.62 MPa
2	300 N/ 15 min	Yes	No	No	No	Yes	No	Yes	Na	3000 N/s	To failure	na	Failure load 3.8–6.5 kN
3	300 N/ 15 min	Yes	Yes	No	Yes	No	No	Yes	Na	0.5°/s	6000 cycles	Axial 1472 N	na
4	300 N/ 15 min	Yes	Yes	Yes	No	No	No	Yes	Na	1000 N/s, 0.5 Hz	Ramp, 1000 cycles	Axial 1000 N	Failure load 10.8 kN
5	na	Yes	Yes	Yes	No	No	No	Yes	17° flex 17° ext	1 mm/s	Ramp	na	Failure load 1.8 kN
6	na	Yes	Yes	Yes	No	No	No	Yes	11° flex 12° ext	1 mm/s	Ramp	na	Failure load 17.7 kN axial, 7.6 kN flex 2.9 kN ext
7	260 N/ 15 min	Yes	Yes	Yes	No	No	No	Yes	Na	45°/s 1 Hz	Max 86,400 cycles	Axial 260/867/1472 N	# cycles to disc failure
8	na	Yes	Yes	Yes	Yes	No	No	Yes	5° flex 5° ext	0.07 Hz	1500 cycles	na	Hysteresis Bending moment 3–3.9 Nm
9	na	Yes	No	No	No	No	No	No	Na	40 N/s	Ramp	Axial 500 N	Disc pressure Max 1.6 MPa
10	300 N/ 15 min	Yes	Yes	Yes	No	Yes	No	Yes	16° flex 16° ext	0.5°/s	Max 10,000 cycles	1500 N	Foramina pressure 6 kPa
11	0, 200, 400 N	Yes	Yes	Yes	Yes	Yes	Yes	Yes	Flex/ext/rot 0.8° lateral 1°	Axial 0.2 mm AP/Lat 0.3 mm	na	Axial 0, 200, 400 N	6 DF Stiffness Linear 0.5–3.5 kN/mm Rotational 2–10 Nm/°
12	300 N/ 15 min	Yes	Yes	Yes	No	No	No	Yes	4.2° flex 6.1° ext	5 Hz	120 min Static	1500 N ± 1250 N Static 1500 N	Stiffness Pre 2.8–2.9 kN/mm Post 2.9–3.0 kN/mm
13	300 N/ 15 min	Yes	No	No	No	No	No	No	na	5 Hz	120 min Static	1400 N ± 140 N Static 1400 N	Modulus 0.3–3.4 MPa Strain 1.3–2.2
14	300 N/ 30 min 1 KN/ 180 min	Yes	Yes	No	No	No	No	Yes	21°	0.5°/s 3000 N/s	To failure	na	Failure load 5.6–12.1 kN
15	500 N/ 30 min	Yes	Yes	Yes	Yes	Yes	Yes	Yes	4° flex. ext. lat	0.1 Hz	60 min	Axial 500 N	Stiffness Matrix 6 DF
16	300 N/ 15 min	Yes	Yes	Yes	No	No	Yes	Yes	7.9° flex 4.4° ext	0.5°/s, 0.05 mm/s	na	15, 30, 45 and 60% of predicted axial failure load	Shear Force 2.2–2.7 kN Shear Stiffness 0.7–1.1 kN/mm
17	300 N/ 15 min	Yes	Yes	Yes	No	No	Yes	Yes	na	0.5°/s 1 Hz	Max 21,600 cycles	Axial 300 N	Shear failure Morphology/Site
18	300 N/ 15 min	Yes	Yes	Yes	No	No	Yes	Yes	15° flex 3.4° ext	0.5°/s 0.2 mm/s	5 cycles	Axial 300 N Shear ± 400 N	Shear Stiffness NZ 58–85 N/mm
19	300 N/ 15 min	Yes	Yes	Yes	No	No	Yes	Yes	na	0.15 mm/s	Ramp	Axial 1546 N ± 22 N	Shear Force 1.9–2.5 kN
20	500 N	Yes	No	No	No	Yes	No	Yes	na	na	30 s	Axial 0,5, 1,0, 1,5 Nm	Vertebral rotation 0.05–1.8°
21	na	Yes	No	No	No	No	No	No	na	5 mm/min	Ramp	na	Failure load 7.9 kN
22	300 N/ 15 min	Yes	No	No	No	No	No	No	na	0.5 Hz	Max 12 h	50, 70, 90% of calc strength	Fatigue # cycles to failure
23	300 N/ 15 min	Yes	Yes	No	No	No	No	Yes	na	0.5 Hz	Max 12 h	10, 30, 50, 70, 90% of calc strength	Fatigue # cycles to failure Injury site
24	300 N/ 15 min	Yes	No	No	No	No	No	No	na	3000 N/s	Ramp	na	Failure strength 10.5 kN
25	na	Yes	Yes	Yes	Yes	No	No	Yes	4° flex 4° lat	1°/s	Step	200–800 N	IVD stress distribution 288–1611 kPa
26	260 N/ 15 min	Yes	Yes	Yes	No	No	No	Yes	15° flex 2° ext	1 Hz	Max 14,400 cycles	Axial 1472 N	Fatigue Failure Injury site
27	na	Yes	No	No	No	No	No	No	na	3 Hz, 5 mm/min	20,000 cycles Ramp	0–1000 N	Failure load 8.3 kN
28	na	Yes	Yes	No	No	No	Yes	Yes	na	0.5 Hz	1500 cycles	Axial 1600 N	Shear strength 1.0–2.4 kN
29	na	Yes	No	No	No	No	Yes	Yes	na	0.1 mm/s	ramp	Axial 1600 N	Shear strength 1.6–2.1 kN
30	300 N/ 15 min	Yes	Yes	Yes	No	No	No	Yes	17° flex 6° ext	0.5 Hz	7000 cycles	Axial 1472 N	Disc herniation Pathway
31	na	Yes	Yes	No	No	No	No	Yes	5°	na	na	Axial 440–1123 N	Flexion stiffness 70–300 Nm/rad
32	na	No	No	No	No	No	No	No	na	na	na	na	Differences in annular mechanical properties in pressurized and un-pressurized discs
33	na	No	No	No	No	No	No	No	na	na	na	na	Fracturing of end-plate as a result of injecting hydraulic solution into IVD
34	300 N/ 15 min	Yes	Yes	Yes	No	No	No	Yes	na	1 Hz	6000 cycles	1260–1540 N	Loss in disc height as a result of compression
35	500 N/ 3 h	Yes	Yes	Yes	Yes	Yes	Yes	No	4°	na	na	na	Obtaining the load–displacement properties of a motion-segment under "physiological conditions"
36	0, 200, 400 N	Yes	Yes	Yes	Yes	Yes	Yes	No	2°	na	87 s	na	Increased preload causes increased stiffness
37	na	Yes	No	No	No	Yes	No	No	na	na	1 h	340 N	Effects of torsion on IVD stress
38	0.001 MPa (IVDP)/ 15 min	Yes	No	No	No	No	No	No	na	na	3 cycles	2,0 MPa (IVDP)	Deformation time-depenency of different FSU-parts under compression
39	20 N/ 15 min	Yes	No	No	No	No	No	No	na	na	3 cycles	Avg 1694 N	IVD height loss after compression
40	na	Yes	Yes	Yes	No	No	No	Yes	na	1.5°/s	7 h	250 N	Neutral zone stiffness after compression
41	na	Yes	No	No	No	Yes	No	Yes	na	na	na	340 N	IVD height loss after compression
42	300 N/ 30 min	Yes	No	No	No	No	No	No	na	na	1 h	1391 N	IVD fluid dynamic during compression
43	300 N/ 15 min	Yes	Yes	Yes	No	No	No	Yes	na	0.5 Hz	12 h max	na	Fatigue test
44	300 N/ 15 min	Yes	No	No	No	No	No	No	na	0.5 Hz	10,800 cycles max	8,3 N/ sec max	Fatigue test
45	300 N/ 15 min	Yes	Yes	Yes	No	No	No	Yes	na	0.5°/s	3 cycles/ na	10 N, 300 N, 600 N and 1200 N	IVD AF bulge change after compression
46	300 N/ 15 min	Yes	Yes	Yes	No	No	No	Yes	na	0.5°/s	na	15% of predicted UCT	Validation of ultrasound to measure mechanical properties during experimentation
47	300 N/ 15 min	Yes	Yes	Yes	No	No	Yes	Yes	na	0.5°/s	na	300 N, 400 N	Facet joint capsule strain durin compression and flexion/extension
48	300 N/ 15 min	Yes	Yes	Yes	No	No	No	Yes	4.3° flex and 5.1° ext	0.5°/s	120 min	1500 N ± 1200 N	IVD height loss, dynamic compressive stiffness
49	300 N/ 15 min	Yes	Yes	Yes	No	No	Yes	Yes	na	1 mm/s, 4 mm/s, 6 mm/s	To failure	300 N, 1600 N	Ultimate anterior shear force, ultimate displacement, average stiffness and energy to failure
50	300 N/ 15 min	Yes	Yes	Yes	No	No	No	Yes	na	0.5°/s	5000 cycles	1500 N	Interfacet spacing
51	300 N/ 15 min	Yes	Yes	Yes	No	No	No	Yes	na	5, 10, 30 cycles/min	5000 cycles	10%, 20% and 40% of UCT	IVD height loss and bulging
52	300 N/ 15 min	Yes	Yes	Yes	No	No	No	Yes	18.3°	45°/s 1 Hz	3600 cycles	1500 N	Axial deformation, IVD pressure change, IVD height change
53	300 N/ 15 min	Yes	Yes	Yes	No	No	No	Yes	na	5, 10, 30 cycles/min	5000 cycles	10%, 20% and 40% of UCT	Damage patterns
54	300 N/ 15 min	Yes	Yes	Yes	No	No	No	Yes	na	0.5°/s	5 cycles	1472 N	Facet-joint fracturing, stiffness
55	300 N/ 15 min	Yes	Yes	Yes	No	No	No	Yes	13.23° flex and 6.23° ext	0.5°/s	7000 cycles	1500 N	IVD herniation
56	na	Yes	No	No	No	No	No	No	na	1700–2500 N/s	To failure	1700–2500 N	Mean ultimate force at failure
57	na	Yes	Yes	Yes	No	No	No	Yes	9–15°	1 Hz	20,000 cycles/ 5.5 h	700 N	Damage patterns on MRI and in histological slices
58	300 N/ 15 min	Yes	No	No	No	No	No	No	na	na	Max 85,000 cycles	1500 N	Mean failure load
59	300 N/ 15 min	Yes	Yes	Yes	No	No	No	Yes	14.8° flex and 4.3° ext	na	10,000 cycles	1500 N	Nucles pulposus migration with flex/ext + compression vs only compression
60	300 N/ 15 min	Yes	Yes	Yes	No	No	No	Yes	na	na	8000 + 8000 cycles	1500 N	Angular stiffness
61	300 N	Yes	No	No	No	No	Yes	No	na	2 Hz	120 min	300 N, 500 N, 600 N, 800 N, 1500 N	Degree of spondylolisthesis and spondylolysis
62	260 N/ 879 s	Yes	Yes	Yes	No	Yes	No	Yes	na	0.5°/s, 45°/s and 1 Hz	na	1472 N	Disc height loss, endplate fracture
63	300 N/ 15 min	Yes	Yes	Yes	No	Yes	No	Yes	12° flex and 6°ext	1 Hz	Max 10,000 cycles	1500 N	Disc herniation
64	300 N/ 15 min	Yes	No	No	No	No	Yes	No	na	100 N/s	To failure	1500 N	Ultimate shear load at failure, deformation at failure, stiffness, energy absorbed
65	300 N/ 15 min	Yes	Yes	No	No	No	No	Yes	10°	100 N/s, 10,810 N/s	To failure	Maximum reported 2345 N	Ultimate load, deformation, energy, stiffness
66	na	Yes	No	No	No	No	Yes	No	na	na	na	na	Damage patterns on MRI and in histological slices
67	500 N/ 15 min	Yes	Yes	Yes	Yes	Yes	No	Yes	na	0.5–5°/s	na	Maximum 500 N	Stiffness
68	500 N/ 3 h	Yes	Yes	Yes	Yes	Yes	No	Yes	na	0,1 Hz, 0,5 Hz	na	500 N	Stiffness Matrix 6 DF
69	300 N/ 15 min	Yes	Yes	Yes	No	No	No	Yes	na	0.5°/s	Min 21,600 cycles to failure	30, 50 and 70% ult compression tolerance	Endplate fatigue failure during cyclic compression loading with variable and consistent peak magnitudes
70	na	No	No	No	No	No	No	No	na	na	na	na	Effect of pressure‐induced fracture on mechanical properties of AF

## Results

The systematic scoping review included 70 studies that had been published between 1997–2021. The included studies are presented in Table 1.

### Specimens

Material information is presented in Table 2. Basic information regarding breed was in general not specified and only mentioned as “domestic” or “landrace” when mentioned. Forty-one (58%) studies mentioned the weight of the pigs, of which 25 (60%) were between 60–80 kg. Thirty-four (65%) studies stated the age of the pigs (some used young/immature), out of which 13 (28%) used pigs that were 4–6 months old. The level of the used FSUs in the included studies were 42 (60%) on cervical, 25 (36%) on lumbar and 1 (1.5%) on thoracic FSU’s.

### Preparation

There were clear similarities in the preparation of the specimens: Fifty (72%) studies had frozen the specimens and then thawed them prior to testing, 51 (72%) kept the specimens moistened during the procedure and 51 (73%) used a preload to reduce post-mortem swelling.

### Load protocols

Loading was done in many ways with varying degrees of reported information (Table 3): Sixty-seven (96%) studies used compressive load or tension, three did not. Forty-four (63%) had an angular load (flexion/extension), out of which only 23 (53%) specified the angle. Load duration and magnitude were heterogenous among the studies. Load protocols ranged from simple one directional compression-tension to multi direction six degrees of freedom (6DF) loadings that required complex lay-out of both test equipment and procedure. A majority of these were performed in custom made testing apparatus or modified material testing machines. Repeated testing in different directions required submaximal loading and the level used varied between the studies but were calculated to be within the apparent linear region of the stress- strain curve or within the physiological range of motion (ROM). Preloading (300–500 N) the specimens for 15 to 180 min were the most common way to counter swelling, but 19 (27%) lacked any information regarding this.

### Study apparatus and validated tests

Sixty-eight (97%) studies mentioned the model of the test-device used, out of which 49 (72%) used an Instron mechanical testing system of model 8511/8872/8874. There was no mention of whether the machine was validated, or when it was last calibrated in any study.

### Biomechanical properties

Table 4 summarizes the mechanical properties in six degrees of freedom, three translations presented as axial shear (often referred to as compression/tension), Lateral shear and A-P shear. Three rotations; sagittal rotation (flexion/extension bending), coronal rotation (lateral bending) and horizontal rotation of the porcine FSU were derived from the articles included in this study. The nomenclature varied in the articles probably due to different scientific traditions. Both alternatives are added in the table to facilitate understanding of it.

**Table 4:** Mechanical properties

Parameter /load mode	Force	Deformation /degrees	Stiffness	Stress range	Strain
Axial compression	0.58—17.0 kN	1.8—6.6 mm	0.5—4.5 kN/mm	0.5—7.7 MPa	na
Axial tension	45—112 N	na	na	na	na
Horizontal rotation	na	0—6°	2.16—10.1 Nm/°	NA	NA
Flexion/extension bending-Sagittal rotation	1.3—92 Nm	3.2—20.5°	0.54—8 .7 Nm/°	NA	NA
Lateral bending-Coronal rotation	na	na	0.63—7 Nm/°	NA	NA
Shear A-P-Lateral	0.3—3.5 kN	0.66—18.8 mm	37—800 N/mm	na	na

*na* not available, *NA* not applicable

## Discussion

The primary result of this study was the conclusion that there is a lack of consensus regarding how the material, methods and results should be documented and presented to achieve comparability and high-quality studies. We found that while many of the included studies used similar test materials when looking at age, weight, and spinal level, very few mentioned the breed of the pig and only as “domestic/landrace”. The spine level used in the included studies varied. Several studies used lumbar vertebrae, but many used cervical vertebrae as displayed in Table 2. There is some evidence that porcine cervical vertebrae is more similar to the human lumbar vertebrae in terms of ROM and morphology as well as failure mechanisms than porcine lumbar vertebrae [16] and is therefore proposed as a good model for lumbar spine studies.

Most studies used similar procedures for preparation, i.e. specimens were kept frozen before use, a pre-load compression to balance swelling was applied and the specimen were kept moisturized during the experiment (Table 3). The preparation of the functional spinal units was in general done in similar style but were also usually reported in general terms. Most of the specimens used were frozen between harvesting and preparation. The literature report divergent findings regarding effects of freezing process. However no or minor impact on the outcome of the study protocol depending on intervention seems to be the general finding [90], however a load rate dependence has been noted [91]. The freeze temperature and storage time were seldom noted, which dependent on study intervention could be important. The thawing time of the specimens was often reported, but in some cases probably underestimated. The importance of a fully thawed specimen that has reached correct study temperature is vital, especially when time-dependent properties are investigated.

The method used to fixate the specimens to the stabilization cups varied among the studies, but the most common practices were by screws, cement such as PMMA or auto body plaster. The fixation methods are generally not validated and are more of a proven experience and how it affects the results are not known. Using a preload to supposedly balance post-mortem swelling of the specimen is conducted in several of the included studies (Table 3), and a study has displayed more in vivo related results compared to no physiological preload [57]. Most of the included studies reported that the specimens were moistened by using a hydrated gauze or similar during the test to counteract de-hydration and thus resemble the normal in situ conditions. This procedure is important [92] but the effect on FSU test results is not clear.

The method and load protocols that were used in the studies were heterogeneous regarding loading time, magnitude, and angle. Nearly every study used a compressive load, with or without an angular load superimposed. Out of the 44 studies that reported using an angular load, only 23 (Table 3) mentioned the specific angle(s) used. Using an angular load but omitting to report angle used makes it difficult to replicate the study, as well as making it impossible to compare it to similar studies. With few exceptions, the load duration and magnitude varied between the studies. Having varied durations and magnitudes between studies with completely different aims is no surprise, but even in those studies with similar aims did it vary.

No included study mentioned whether the technical equipment used in the experiment was validated, and none mentioned when the loading system was last calibrated or if a direct calibration using calibration weights and lengths is performed. Using a validated system would improve the evidence and quality provided by the study.

Load rate nomenclature was dependent on load mode, and expressed as force or stress rate, deformation or strain rate and torque rate. This varied between the studies, mainly because of different research questions. If appropriate parameters are reported, a transformation of load rate is feasible, making a comparison between studies possible. A conformity to a use of SI units would facilitate interpretation of data as well as simplify comparison between studies and is highly recommended.

To achieve an overall estimate of the mechanical properties presented, we chose to present range rather than mean and standard deviation since the values are derived from studies with inter varying loading pre-requisitions, sometimes the only common factor being the load mode or direction. Axial compression testing mode seems to be the most common loading mode in the articles as opposed to axial tension where there was insufficient information. These overall findings can aid in the layout of future studies necessary for adding knowledge about the loading mechanism of porcine FSU.

### Strengths and limitations

#### Selection and systematic bias

The search and selection process of search criteria was done through a stepwise process and addressed the MESH terms and included all useful synonyms available. The database search was completed with an author search to achieve less systematic drop out in the selection. The manual selection process of the studies was not validated but was done in a controlled manner where all studies were analyzed by several of the authors according to the preset protocol.

A review based on additional animal species (such as calf, sheep, and dogs) would enhance the overall knowledge regarding how animal models are used in spinal research, how these studies report basic parameters regarding material and methods and thereby increase the external validity of the current study. This scoping review aimed to primarily address the field of porcine FSU to achieve higher quality in the methodology to achieve higher internal validity but with the potential limitation of external validity. Different animal models have different material properties and the use of porcine specimens in spine research has been widely accepted for many years but is highly dependent on research questions. Anatomical and ROM similarities between cervical porcine FSU and human lumbar FSU indicate that the porcine cervical FSU is a reasonably good model for research questions regarding ROM in the human lumbar spine [4–6, 16]. The present study did only include non-operated and non-instrumented FSUs that further reduced the available material but did enhance the possibility to compare the research results of basic loading parameters. Operated and instrumented specimens are intervened which may affect the basic loading parameters and the biomechanical properties of the FSU. Multisegmented spines were also excluded due to the difference in ROM and other loading parameters compared to FSU.

#### Publication bias

All included studies have been published in peer reviewed journals according to Table 1 and indexed in the Scopus or PubMed databases.

### Clinical use and significance

This systematic scoping review highlight the importance to increase the scientific evidence level and quality in porcine FSU spinal research. We suggest that the results from this systematic scoping review may grant a better understanding of how future studies should be best conducted to present valid, reliable, and comparable data, which in turn may bring us closer to understanding the physical boundaries of the spine and to reduce unnecessary animal experimentation.

### Ethical considerations

The usage of pigs for animal experimentation constitutes an ethical problem and means to minimize the number of animals used is a priority. One way could be to define a common accepted research protocol for in vitro spinal biomechanical testing. The similarities between the spinal properties of the pig compared to that of humans, is believed to be great enough to make it possible to draw parallels between the results from such studies with human biomechanical properties and thus justify them.

### Future considerations and study protocol suggestion

Our study shows the importance of comprehensive reporting of relevant data concerning material, method, and methods of validation in experimental animal studies.

We suggest that future studies increase the information in the reports regarding study material and to validate the study method to enhance the internal and external validity of the study. We suggest that future study reports are based on the ARRIVE Guidelines [2] and the following basic template:

- **Material:**
  - Detailed material information (breed, weight, age etc.).
  - Physical size of test material such as vertebral diameter and disc height
  - Standardization and validation of material loading parameters, through compression to failure of one single included specimen
  - Pre-test handling and preparation such as report of harvest, storage (temperature, time) and fixation to the testing equipment.
- **Test conditions:**
  - Environmental conditions, temperature etc.
  - Material conditioning, for example, means to minimize de-hydration.
- **Test apparatus validation**
  - Report of test apparatus
  - Report of validation of test apparatus
- **Test protocol**
  - Preload
  - Defined and reported load, time, frequency, angle and test protocol variations.
  - Validated test protocol

## Conclusion

Biomechanical testing on FSU units is a commonly used experimental spine research procedure. A notable variability in the amount of information that is reported in the materials and method section in the articles was identified in this review. A basic research guideline regarding improved report-structure, that would enable comparison between biomechanical experimental studies and increase the method quality, is presented in the present study. It is also evident that there is a clear need for a validated quality-assessment template for experimental animal studies. 

## References

[1] Hartvigsen J, Hancock MJ, Kongsted A, Louw Q, Ferreira ML, Genevay S (2018). What low back pain is and why we need to pay attention. Lancet.

[2] Kilkenny C, Browne WJ, Cuthill IC, Emerson M, Altman DG (2010). Improving bioscience research reporting: the ARRIVE guidelines for reporting animal research. PLoS Biol.

[3] Smit TH (2002). The use of a quadruped as an in vivo model for the study of the spine - biomechanical considerations. Eur Spine J.

[4] Wilke HJ, Geppert J, Kienle A (2011). Biomechanical in vitro evaluation of the complete porcine spine in comparison with data of the human spine. Eur Spine J.

[5] Wilke HJ, Kettler A (1997). Claes LE Are sheep spines a valid biomechanical model for human spines?. Spine(Phila Pa 1976).

[6] Wilke HJ, Krischak S, Claes L (1996). Biomechanical comparison of calf and human spines. J Orthop Res.

[7] Wilke HJ, Rohlmann A, Neller S, Graichen F, Claes L (2003). Bergmann G ISSLS prize winner: A novel approach to determine trunk muscle forces during flexion and extension: a comparison of data from an in vitro experiment and in vivo measurements. Spine(Phila Pa 1976).

[8] Alini M, Eisenstein SM, Ito K, Little C, Kettler AA, Masuda K (2008). Are animal models useful for studying human disc disorders/degeneration?. Eur Spine J.

[9] Beckstein JC, Sen S, Schaer TP, Vresilovic EJ (2008). Elliott DM Comparison of animal discs used in disc research to human lumbar disc: axial compression mechanics and glycosaminoglycan content. Spine(Phila Pa 1976).

[10] Lotz JC (2004). Animal models of intervertebral disc degeneration: lessons learned. Spine(Phila Pa 1976).

[11] Lundin O, Ekstrom L, Hellstrom M, Holm S, Sward L (2000). Exposure of the porcine spine to mechanical compression: differences in injury pattern between adolescents and adults. Eur Spine J.

[12] Lysack JT, Dickey JP, Dumas GA, Yen D (2000). A continuous pure moment loading apparatus for biomechanical testing of multi-segment spine specimens. J Biomech.

[13] Showalter BL, Beckstein JC, Martin JT, Beattie EE, Espinoza Orias AA, Schaer TP (2012). Comparison of animal discs used in disc research to human lumbar disc: torsion mechanics and collagen content. Spine(Phila Pa 1976).

[14] Tsai KH, Chang GL, Lin RM (1997). Differences in mechanical response between fractured and non-fractured spines under high-speed impact. Clin Biomech (Bristol, Avon).

[15] van Deursen DL, Snijders CJ, Kingma I (2001). van Dieën JH In vitro torsion-induced stress distribution changes in porcine intervertebral discs. Spine(Phila Pa 1976).

[16] Yingling VR, Callaghan JP, McGill SM (1999). The porcine cervical spine as a model of the human lumbar spine: an anatomical, geometric, and functional comparison. J Spinal Disord.

[17] Baranto A, Ekstrom L, Hellstrom M, Lundin O, Holm S (2005). Sward L Fracture patterns of the adolescent porcine spine: an experimental loading study in bending-compression. Spine(Phila Pa 1976).

[18] Thoreson O, Baranto A, Ekstrom L, Holm S, Hellstrom M, Sward L (2010). The immediate effect of repeated loading on the compressive strength of young porcine lumbar spine. Knee Surg Sports Traumatol Arthrosc.

[19] Arksey H, O’Malley L (2005) Scoping studies: towards a methodological framework. Int J Soc Res Methodol 8:19–32

[20] Peters MD, Godfrey CM, Khalil H, McInerney P, Parker D, Soares CB (2015). Guidance for conducting systematic scoping reviews. Int J Evid Based Healthc.

[21] de Vries RBM, Hooijmans CR, Langendam MW, van Luijk J, Leenaars M, Ritskes-Hoitinga M, et al. (2015) A protocol format for the preparation, registration and publication of systematic reviews of animal intervention studies. Evid Based Preclinical Med 2:e00007. ISSN 2054-703X

[22] Tricco AC, Lillie E, Zarin W, O’Brien KK, Colquhoun H, Levac D et al (2018) PRISMA Extension for Scoping Reviews (PRISMA-ScR): Checklist and Explanation. Ann Intern Med 169:467–473 10.7326/M18-0850 30178033

[23] Page MJ, McKenzie JE, Bossuyt PM, Boutron I, Hoffmann TC, Mulrow CD (2021). The PRISMA 2020 statement: an updated guideline for reporting systematic reviews. BMJ.

[24] Araujo AR, Peixinho N, Pinho AC, Claro JC (2015). Quasi-static and dynamic properties of the intervertebral disc: experimental study and model parameter determination for the porcine lumbar motion segment. Acta Bioeng Biomech.

[25] Aultman CD, Drake JD, Callaghan JP (2004). McGill SM The effect of static torsion on the compressive strength of the spine: an in vitro analysis using a porcine spine model. Spine(Phila Pa 1976).

[26] Aultman CD, Scannell J, McGill SM (2005). The direction of progressive herniation in porcine spine motion segments is influenced by the orientation of the bending axis. Clin Biomech (Bristol, Avon).

[27] Balkovec C, McGill S (2012). Extent of nucleus pulposus migration in the annulus of porcine intervertebral discs exposed to cyclic flexion only versus cyclic flexion and extension. Clin Biomech (Bristol, Avon).

[28] Baranto A, Ekstrom L, Holm S, Hellstrom M, Hansson HA, Sward L (2005). Vertebral fractures and separations of endplates after traumatic loading of adolescent porcine spines with experimentally-induced disc degeneration. Clin Biomech (Bristol, Avon).

[29] Callaghan JP, McGill SM (2001). Intervertebral disc herniation: studies on a porcine model exposed to highly repetitive flexion/extension motion with compressive force. Clin Biomech (Bristol, Avon).

[30] Chow DH, Luk KD, Holmes AD, Li XF, Tam SC (2004). Multi-planar bending properties of lumbar intervertebral joints following cyclic bending. Clin Biomech (Bristol, Avon).

[31] Dennison CR, Wild PM, Dvorak MF, Wilson DR (2008). Cripton PA Validation of a novel minimally invasive intervertebral disc pressure sensor utilizing in-fiber Bragg gratings in a porcine model: an ex vivo study. Spine(Phila Pa 1976).

[32] Drake JD, Callaghan JP (2009). Intervertebral neural foramina deformation due to two types of repetitive combined loading. Clin Biomech (Bristol, Avon).

[33] Gardner-Morse MG, Stokes IA (2003). Physiological axial compressive preloads increase motion segment stiffness, linearity and hysteresis in all six degrees of freedom for small displacements about the neutral posture. J Orthop Res.

[34] Gooyers CE, McMillan RD, Howarth SJ (2012). Callaghan JP The impact of posture and prolonged cyclic compressive loading on vertebral joint mechanics. Spine(Phila Pa 1976).

[35] Gregory DE, Callaghan JP (2012). An examination of the mechanical properties of the annulus fibrosus: the effect of vibration on the intra-lamellar matrix strength. Med Eng Phys.

[36] Gunning JL, Callaghan JP, McGill SM (2001). Spinal posture and prior loading history modulate compressive strength and type of failure in the spine: a biomechanical study using a porcine cervical spine model. Clin Biomech (Bristol, Avon).

[37] Holsgrove TP, Gill HS, Miles AW, Gheduzzi S (2015). The dynamic, six-axis stiffness matrix testing of porcine spinal specimens. Spine J.

[38] Howarth SJ, Callaghan JP (2012). Compressive force magnitude and intervertebral joint flexion/extension angle influence shear failure force magnitude in the porcine cervical spine. J Biomech.

[39] Howarth SJ, Callaghan JP (2013). Towards establishing an occupational threshold for cumulative shear force in the vertebral joint - an in vitro evaluation of a risk factor for spondylolytic fractures using porcine specimens. Clin Biomech (Bristol, Avon).

[40] Howarth SJ, Gallagher KM, Callaghan JP (2013). Postural influence on the neutral zone of the porcine cervical spine under anterior-posterior shear load. Med Eng Phys.

[41] Howarth SJ, Giangregorio LM, Callaghan JP (2013). Development of an equation for calculating vertebral shear failure tolerance without destructive mechanical testing using iterative linear regression. Med Eng Phys.

[42] Kouwenhoven JW, Smit TH, van der Veen AJ, Kingma I, van Dieen JH (2007). Castelein RM Effects of dorsal versus ventral shear loads on the rotational stability of the thoracic spine: a biomechanical porcine and human cadaveric study. Spine(Phila Pa 1976).

[43] Lundin O, Ekstrom L, Hellstrom M, Holm S (1998). Sward L Injuries in the adolescent porcine spine exposed to mechanical compression. Spine(Phila Pa 1976).

[44] Parkinson RJ, Callaghan JP (2007). Can periods of static loading be used to enhance the resistance of the spine to cumulative compression?. J Biomech.

[45] Parkinson RJ, Callaghan JP (2009). The role of dynamic flexion in spine injury is altered by increasing dynamic load magnitude. Clin Biomech (Bristol, Avon).

[46] Parkinson RJ, Durkin JL (2005). Callaghan JP Estimating the compressive strength of the porcine cervical spine: an examination of the utility of DXA. Spine(Phila Pa 1976).

[47] Ryan G, Pandit A, Apatsidis D (2008). Stress distribution in the intervertebral disc correlates with strength distribution in subdiscal trabecular bone in the porcine lumbar spine. Clin Biomech (Bristol, Avon).

[48] Tampier C, Drake JD, Callaghan JP (2007). McGill SM Progressive disc herniation: an investigation of the mechanism using radiologic, histochemical, and microscopic dissection techniques on a porcine model. Spine(Phila Pa 1976).

[49] van Dieen JH, van der Veen A, van Royen BJ (2006). Kingma I Fatigue failure in shear loading of porcine lumbar spine segments. Spine(Phila Pa 1976).

[50] van Solinge GB, van der Veen AJ, van Dieen JH, Kingma I, van Royen BJ (2010). Anterior shear strength of the porcine lumbar spine after laminectomy and partial facetectomy. Eur Spine J.

[51] Yates JP, Giangregorio L (2010). McGill SM The influence of intervertebral disc shape on the pathway of posterior/posterolateral partial herniation. Spine(Phila Pa 1976).

[52] Zondervan RL, Popovich JM, Radcliffe CJ, Pathak PK, Reeves NP (2016). Sagittal rotational stiffness and damping increase in a porcine lumbar spine with increased or prolonged loading. J Biomech.

[53] Ghelani RN, Zwambag DP, Gregory DE (2020). Rapid increase in intradiscal pressure in porcine cervical spine units negatively impacts annulus fibrosus strength. J Biomech.

[54] Brown SH, Gregory DE, McGill SM (2008). Vertebral end-plate fractures as a result of high rate pressure loading in the nucleus of the young adult porcine spine. J Biomech.

[55] Gregory DE (2011). Callaghan JP Does vibration influence the initiation of intervertebral disc herniation? An examination of herniation occurrence using a porcine cervical disc model. Spine(Phila Pa 1976).

[56] Stokes IA, Gardner-Morse M, Churchill D, Laible JP (2002). Measurement of a spinal motion segment stiffness matrix. J Biomech.

[57] Gardner-Morse MG, Stokes IA, Churchill D, Badger G (2002). Motion segment stiffness measured without physiological levels of axial compressive preload underestimates the in vivo values in all six degrees of freedom. Stud Health Technol Inform.

[58] van der Veen AJ, Mullender MG, Kingma I, van Dieen JH, Smit TH (2008). Contribution of vertebral [corrected] bodies, endplates, and intervertebral discs to the compression creep of spinal motion segments. J Biomech.

[59] van der Veen AJ, Mullender M, Smit TH, Kingma I (2005). van Dieën JH Flow-related mechanics of the intervertebral disc: the validity of an in vitro model. Spine(Phila Pa 1976).

[60] Smit TH, van Tunen MS, van der Veen AJ, Kingma I, van Dieën JH (2011). Quantifying intervertebral disc mechanics: a new definition of the neutral zone. BMC Musculoskelet Disord.

[61] van Deursen DL, Snijders CJ, van Dieën JH, Kingma I, van Deursen LL (2001). The effect of passive vertebral rotation on pressure in the nucleus pulposus. J Biomech.

[62] Kingma I, Weinans H, van Dieën JH, de Boer RW (1998). Finite element aided tracking of signal intensity changes in deforming intervertebral disc tissue. Magn Reson Imaging.

[63] Zehr JD, Tennant LM, Callaghan JP (2019). Incorporating loading variability into in vitro injury analyses and its effect on cumulative compression tolerance in porcine cervical spine units. J Biomech.

[64] Zehr JD, Buchman-Pearle JM, Callaghan JP (2020). Joint fatigue-failure: A demonstration of viscoelastic responses to rate and frequency loading parameters using the porcine cervical spine. J Biomech.

[65] Fewster KM, Noguchi M, Gooyers CE, Wong A, Callaghan JP (2020). Exploring the regional disc bulge response of the cervical porcine intervertebral disc under varying loads and posture. J Biomech.

[66] McKinnon CD, Callaghan JP (2019). Validation of an Ultrasound Protocol to Measure Intervertebral Axial Twist during Functional Twisting Movements in Isolated Functional Spinal Units. Ultrasound Med Biol.

[67] Zehr JD, Barrett JM, Fewster KM, Laing AC, Callaghan JP (2020). Strain of the facet joint capsule during rotation and translation range-of-motion tests: an in vitro porcine model as a human surrogate. Spine J.

[68] Barrett JM, Gooyers CE, Karakolis T, Callaghan JP (2016) The Impact of Posture on the Mechanical Properties of a Functional Spinal Unit During Cyclic Compressive Loading. J Biomech Eng 138(8):081007. 10.1115/1.4033916 10.1115/1.4033916 27322199

[69] Gallagher KM, Howarth SJ, Callaghan JP (2010). Effects of anterior shear displacement rate on the structural properties of the porcine cervical spine. J Biomech Eng.

[70] Drake JD, Dobson H (2008). Callaghan JP The influence of posture and loading on interfacet spacing: an investigation using magnetic resonance imaging on porcine spinal units. Spine(Phila Pa 1976).

[71] Gooyers CE, Callaghan JP (2015). Exploring interactions between force, repetition and posture on intervertebral disc height loss and bulging in isolated porcine cervical functional spinal units from sub-acute-failure magnitudes of cyclic compressive loading. J Biomech.

[72] Noguchi M, Gooyers CE, Karakolis T, Noguchi K, Callaghan JP (2016). Is intervertebral disc pressure linked to herniation?: An in-vitro study using a porcine model. J Biomech.

[73] Gooyers CE, McMillan EM, Noguchi M, Quadrilatero J, Callaghan JP (2015). Characterizing the combined effects of force, repetition and posture on injury pathways and micro-structural damage in isolated functional spinal units from sub-acute-failure magnitudes of cyclic compressive loading. Clin Biomech (Bristol, Avon).

[74] Drake JD, Aultman CD, McGill SM, Callaghan JP (2005). The influence of static axial torque in combined loading on intervertebral joint failure mechanics using a porcine model. Clin Biomech (Bristol, Avon).

[75] Yates JP (2011). McGill SM The effect of vibration and posture on the progression of intervertebral disc herniation. Spine(Phila Pa 1976).

[76] Lundin O, Ekström L, Hellström M, Holm S, Swärd L (2000). Exposure of the porcine spine to mechanical compression: differences in injury pattern between adolescents and adults. Eur Spine J.

[77] Thoreson O, Ekström L, Hansson HA, Todd C, Witwit W, Swärd Aminoff A (2017). The effect of repetitive flexion and extension fatigue loading on the young porcine lumbar spine, a feasibility study of MRI and histological analyses. J Exp Orthop.

[78] Bateman AH, Balkovec C, Akens MK, Chan AH, Harrison RD, Oakden W (2016). Closure of the annulus fibrosus of the intervertebral disc using a novel suture application device-in vivo porcine and ex vivo biomechanical evaluation. Spine J.

[79] Balkovec C, Vernengo J, McGill SM (2013). The use of a novel injectable hydrogel nucleus pulposus replacement in restoring the mechanical properties of cyclically fatigued porcine intervertebral discs. J Biomech Eng.

[80] Beadon K, Johnston JD, Siggers K, Itshayek E (2008). Cripton PA A repeatable ex vivo model of spondylolysis and spondylolisthesis. Spine(Phila Pa 1976).

[81] Scannell JP (2009). McGill SM Disc prolapse: evidence of reversal with repeated extension. Spine(Phila Pa 1976).

[82] Marshall LW, McGill SM (2010). The role of axial torque in disc herniation. Clin Biomech (Bristol, Avon).

[83] Yingling VR, McGill SM (1999). Mechanical properties and failure mechanics of the spine under posterior shear load: observations from a porcine model. J Spinal Disord.

[84] Yingling VR (1999). McGill SM Anterior shear of spinal motion segments. Kinematics, kinetics, and resultant injuries observed in a porcine model. Spine(Phila Pa 1976).

[85] McGill SM, Yingling VR (1999). Traction may enhance the imaging of spine injuries with plane radiographs: implications for the laboratory versus the clinic. Clin Biomech (Bristol, Avon).

[86] Holsgrove TP, Miles AW, Gheduzzi S (2017). The application of physiological loading using a dynamic, multi-axis spine simulator. Med Eng Phys.

[87] Holsgrove TP, Gheduzzi S, Gill HS, Miles AW (2014). The development of a dynamic, six-axis spine simulator. Spine J.

[88] Zehr JD, Tennant LM, Callaghan JP (2019). Examining endplate fatigue failure during cyclic compression loading with variable and consistent peak magnitudes using a force weighting adjustment approach: an in vitro study. Ergonomics.

[89] Snow CR, Harvey-Burgess M, Laird B, Brown SHM, Gregory DE (2018). Pressure-induced end-plate fracture in the porcine spine: Is the annulus fibrosus susceptible to damage?. Eur Spine J.

[90] Azarnoosh M, Stoffel M, Quack V, Betsch M, Rath B, Tingart M (2017). A comparative study of mechanical properties of fresh and frozen-thawed porcine intervertebral discs in a bioreactor environment. J Mech Behav Biomed Mater.

[91] Callaghan JP, McGill SM (1995). Frozen storage increases the ultimate compressive load of porcine vertebrae. J Orthop Res.

[92] Gruevski KM, Gooyers CE, Karakolis T, Callaghan JP (2016) The Effect of Local Hydration Environment on the Mechanical Properties and Unloaded Temporal Changes of Isolated Porcine Annular Samples. J Biomech Eng 138(10):104502 10.1115/1.4034335 27479500

