# Correction: Porcine Functional Spine Unit in orthopedic research, a systematic scoping review of the methodology

**DOI:** 10.1186/s40634-022-00505-8

**Published:** 2022-07-18

**Authors:** Jacob Hedlund, Lars Ekström, Olof Thoreson

**Affiliations:** 1 Department of Orthopedics, Institute of Clinical Sciences at Sahlgrenska Academy, University of Gothenburg, Gothenburg, Sweden; 2 Orthopaedic Research Unit, Sahlgrenska University Hospital, Mölndal, Sweden; 3 Research and Development Primary Health Care, R&D Centre Gothenburg and Södra Bohuslän, Gothenburg, Sweden

**Correction: J Exp Ortop 9, 54 (2022)**

10.1186/s40634-022-00488-6

In the original publication of this article [1], the abstract was missing.

**Absract**

**Purpose**

The aim of this study was to conduct a systematic scoping review of previous in vitro spine studies that used pig functional spinal units (FSU) as a model to gain an understanding of how different experimental methods are presented in the literature. Research guidelines are often used to achieve high quality in methods, results, and reports, but no research guidelines are available regarding in vitro biomechanical spinal studies.

**Methods**

A systematic scoping review approach and protocol was used for the study with a systematic search in several data bases combined with an extra author search. The articles were examined in multiple stages by two different authors in a blinded manner. Data was extracted from the included articles and inserted into a previously crafted matrix with multiple variables. The data was analyzed to evaluate study methods and quality and included 70 studies.

**Results**

The results display that there is a lack of consensus regarding how the material, methods and results are presented. Load type, duration and magnitude were heterogeneous among the studies, but sixty-seven studies (96%) did include compressive load or tension in the testing protocol.

**Conclusions**

This study concludes that an improvement of reported data in the present field of research is needed. A protocol, modified from the ARRIVE guidelines, regarding enhanced report-structure, that would enable comparison between studies and improve the method quality is presented in the current study. There is also a clear need for a validated quality-assessment template for experimental animal studies.

The original article [1] has been corrected.
